# State of the art in biosafety at the European National Reference Laboratories for Transmissible Spongiform Encephalopathies

**DOI:** 10.3389/fpubh.2025.1733350

**Published:** 2026-02-10

**Authors:** M. Begovoeva, R. Nonno, M. Mazza, E. Bozzetta, G. Ru, Colleagues of the European Network of National Reference Laboratories for Transmissible Spongiform Encephalopathies

**Affiliations:** 1 Istituto Zooprofilattico Sperimentale del Piemonte, Liguria e Valle d'Aosta, Torino, Italy; 2 Istituto Superiore di Sanità, Roma, Italy

**Keywords:** biosafety, BSE, CJD, occupational risk, prions, TSE

## Abstract

**Introduction:**

Laboratory technicians handling material containing or potentially infected with prions are exposed to the occupational risk of contracting infectious forms of Creutzfeldt–Jakob disease. The occurrence of three fatalities among laboratory personnel with a history of occupational exposure to bovine spongiform encephalopathy has highlighted the need for greater attention to the correct implementation of biosafety procedures in laboratories that handle prions.

**Methods:**

The European Reference Laboratory for Transmissible Spongiform Encephalopathies (EURL-TSE) carried out a survey to review biosafety procedures currently adopted by the 31 National Reference Laboratories for TSEs (NRLs), stimulate discussion among laboratory directors, and define future actions aimed at reducing the risk of occupational exposure among laboratory personnel.

**Results:**

The survey showed that 83.9% of laboratories adhered to TSE-specific biosafety guidelines, and 61.3% had conducted a biosafety risk assessment, while the biosafety level of the facilities had been determined in 93.5% of cases. In addition, 83.9% reported implementing staff biosafety training protocols, and 77.4% had procedures addressing the risk associated with sharp or pointed tools. Most laboratories had established biosafety procedures for ELISA and western blot assays, whereas fewer had protocols for pathology and immunohistochemistry. Among the three facilities performing experimental inoculations, only two indicated having specific biosafety measures in place.

**Discussion:**

Despite the overall positive results, the survey showed that biosafety procedures are applied heterogeneously across the NRLs. The EURL-TSE will further encourage the full implementation of specific biosafety procedures to address the gaps identified by the survey. The EURL-TSE has created a digital repository of guidelines and bibliographical sources on the subject. It also initiated biannual virtual meetings to discuss critical issues emerged from the survey and to encourage the mutual exchange of experiences and good practices between NRLs Directors.

## 1 Introduction

Transmissible spongiform encephalopathies (TSEs) are fatal neurodegenerative disorders affecting humans and several other mammal species. These conditions are caused by the accumulation, mainly in the central nervous system, of misfolded prion proteins. This accumulation generates lesions in the encephalic tissue and thus confers a spongiform appearance (1). Creutzfeldt–Jakob disease (CJD) is the most common human TSE. Based on its aetiology, CJD is classified in sporadic (sCJD), genetic (gCGD) and infectious forms, the latter resulting from transmission between humans or from animals and including iatrogenic CJD (iCJD) and variant CJD (vCJD) (2). Major non-human TSEs are Bovine Spongiform Encephalopathy (BSE) in cattle, chronic wasting disease (CWD) in cervids, and scrapie in sheep and goats. Both human and animal prion diseases are characterized by a long incubation period followed by the onset of rapidly progressive neurological symptoms and an invariably fatal outcome, as there are no treatments available (2). Given the absence of immune system involvement, to date it is not possible to make a reliable intravitam diagnosis of these pathologies. Moreover, due to their lack of nucleic acids and proteinaceous nature, the prions show high resistance to decontamination procedures commonly used against other microorganisms, posing a serious threat to laboratory personnel and requiring the careful implementation of appropriate risk management strategies (3). Health professionals are exposed to the risk of contracting infectious forms of CJD. Humans can in fact contract iCJD when exposed to CJD-infected material, or vCJD if exposed to BSE prions (2). BSE transmission from animals to humans through the food chain has unfortunately long since been confirmed and associated to vCJD cases, while this possibility has not yet been ruled out for CWD (4). Although some experimental studies have suggested that specific strains of scrapie may cause forms of human CJD with clinical and pathological features indistinguishable from sCJD (5, 6), no epidemiological evidence has yet supported this hypothesis. Even though no relative excess of sCJD was found among healthcare professionals, limitations in registries and case-control studies preclude the definitive exclusion of this possibility (7). Furthermore, this finding does not rule out the potential for specific professions to be associated with an occupational risk (7), as in the case of laboratory technicians handling material containing prions or potentially infected with them.

Biosafety is the implementation of principles, technologies and practices designed to prevent unintended exposure to biological agents or their accidental release (3, 8). Despite the existence of laboratory biosafety guidelines, the severe threat posed by prions prompted the development of several studies and the drafting of specific TSE biosafety guidelines by national and international institutions. Nevertheless, three cases of vCJD have been documented in laboratory personnel with occupational exposure to TSE agents. In Italy, a patient affected by variant CJD who died in 2016 had worked with brain tissue infected by BSE, even though no laboratory accidents were reported in this regard (9). Two cases were described in France in 2020 and 2021, both involving laboratory workers who had experienced accidental occupational exposure 7.5 and 15 years before the onset of clinical symptoms, respectively (9, 10).

The notification of these occupational fatalities has been of great concern to the operators working in the field of prion diseases. In response to these concerns, the staff of the European Reference Laboratory for TSEs (EURL-TSE) highlighted the need for a comprehensive review of the biosafety procedures of the European National Reference Laboratories for TSEs (NRLs). To this end, in September 2023, the EURL-TSE carried out a survey to collect detailed information about the procedures adopted by each of the NRLs. The aim of the initiative was to promote the harmonization and potential implementation of biosafety measures through benchmarking activities and discussions at scheduled meetings. This paper presents the findings of the survey and describes follow-up actions undertaken by the EURL-TSE.

## 2 Materials and methods

The WHO laboratory biosafety guidelines (11) were used as a reference to design a standardised online questionnaire (see Supplementary material) which was administered to the Directors of the 31 NRLs (27 European NRLs plus Iceland, Norway and Switzerland, Italy was represented twice, as distinct replies were collected by the focal points of the two institutions that constitute the Italian NRL).

The questionnaire consisted of 25 items, including 21 binary (Yes/No) questions assessing the implementation of biosafety procedures, two multiple-choice questions addressing types of laboratory activities performed and risk-based segregation of activities, and two open-ended questions regarding the laboratory’s assigned biosafety level and any additional comments. Content validity was ensured through internal review against the WHO laboratory biosafety guidelines to confirm coverage of all relevant aspects of laboratory biosafety. Internal piloting was conducted among the survey team members to assess question clarity, appropriateness, and relevance.

Responses were summarized using descriptive statistics calculated on the overall dataset. Activities conducted and compliance with biosafety procedures were stratified by biosafety level (BSL). Data on the availability of specific biosafety procedures for ELISA/Western Blot, pathology/immunohistochemistry, and experimental inoculations were stratified by laboratory activity (diagnosis, research, or experimental inoculations) to assess whether the implementation of biosafety procedures varied according to the type of activity performed. Descriptive statistics and figures were generated in R (version 4.4.2; R Core Team, 2024), primarily using the *ggplot2* package.

Participation in the survey was voluntary. Respondents were asked to provide their name and institutional affiliation solely to ensure accurate representation of each NRL, and they were informed that all results would be analysed and reported in an anonymised format. Personal identifiers were used only for administrative purposes, stored securely with access restricted to the survey coordinators, and handled in accordance with the General Data Protection Regulation (GDPR) and the data protection requirements of the coordinating institution (EURL-TSE, Istituto Zooprofilattico Sperimentale del Piemonte, Liguria e Valle d’Aosta). Before analysis, all personal identifiers were removed, and a fully anonymised dataset was used. As the study involved anonymised institutional data and did not include sensitive personal information, no additional ethical approvals were required.

## 3 Results

**Figure 1:**
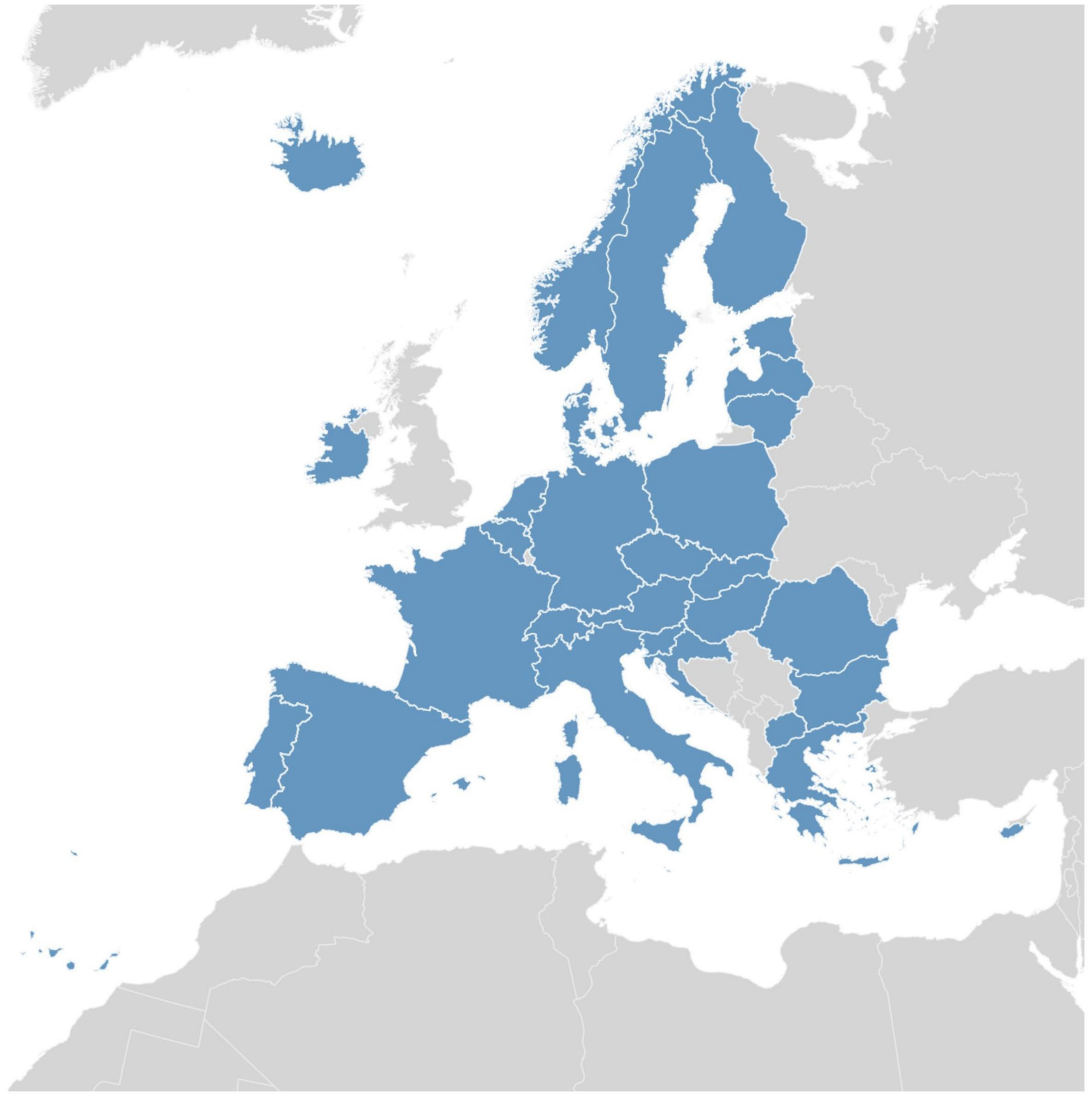
Map showing the countries hosting the 31 surveyed NRLs highlighted in blue.

The survey achieved a 100.0% response rate, with feedback received from all 31 NRL Directors contacted (Figure 1). The countries hosting the 31 surveyed NRLs were Austria, Belgium, Bulgaria, Croatia, Cyprus, Czechia, Denmark, Estonia, Finland, France, Germany, Greece, Hungary, Iceland, Ireland, Italy, Latvia, Lithuania, Malta, The Netherlands, North Macedonia, Norway, Poland, Portugal, Romania, Slovakia, Slovenia, Spain, Sweden, Switzerland.

**Figure 2:**
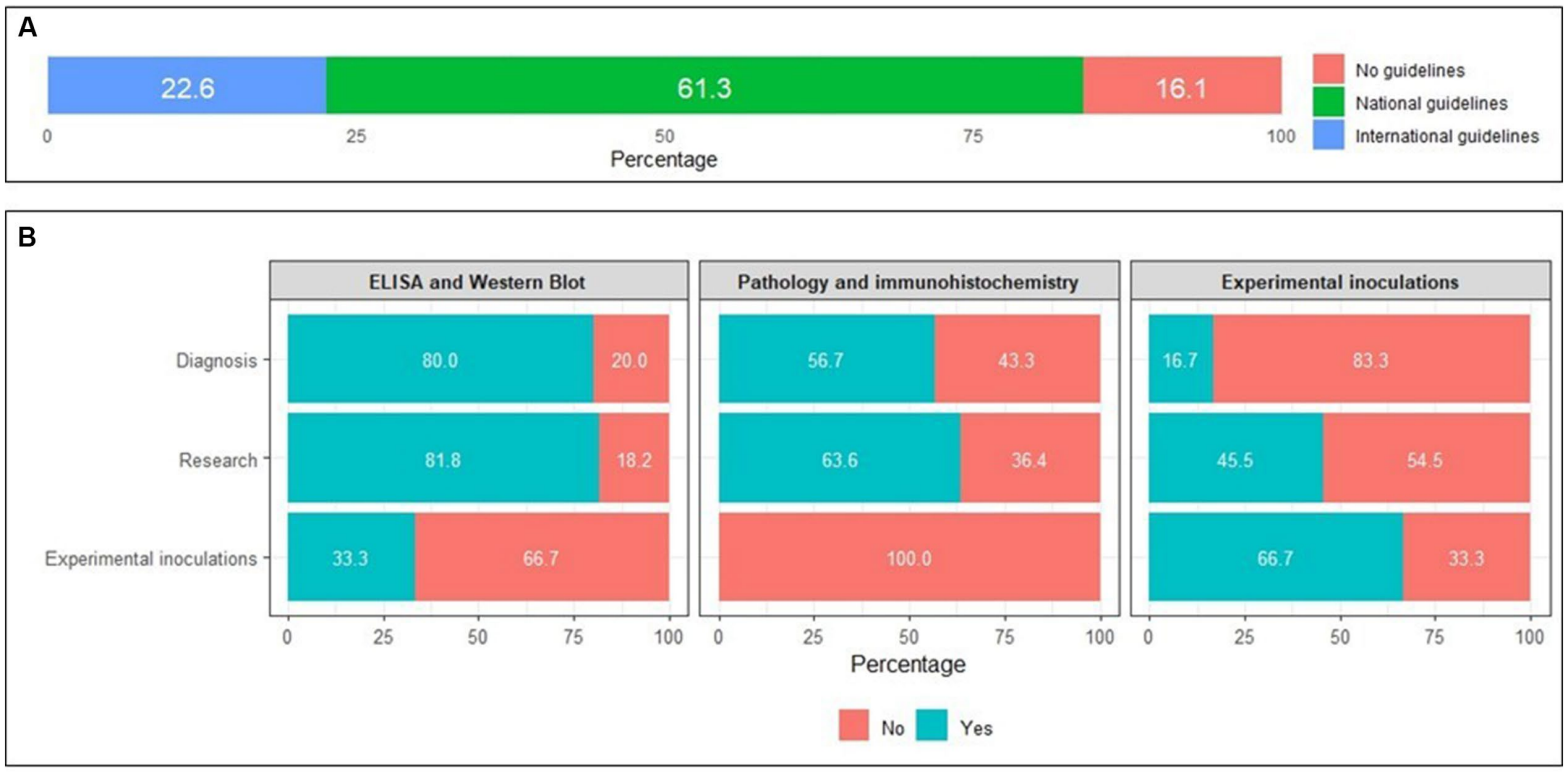
**(A)** Percentages of National Reference Laboratories applying specific guidelines for safely working with transmissible spongiform encephalopathy agents; **(B)** Availability of specific biosafety procedures for selected laboratory techniques, stratified by types of activity conducted by the laboratory (diagnosis, research, experimental inoculations).

Most NRLs (*n* = 26, 83.9%) adhered to specific guidelines for safely working with TSEs, with three quarters of these relying on national guidelines (*n* = 19, 61.3%) and the remaining quarter referring to the international sources listed in Table 1 (*n* = 7, 22.6%) (Figure 2A).

**Table 1:** Resources employed as biosafety guidelines by National Reference Laboratories that do not use specific national guidelines for transboundary spongiform encephalopathies.

Resource	Author	Type of resource	Respondents referencing the resource	Reference
Manual of Diagnostic Tests and Vaccines for Terrestrial Animals	World Organisation for Animal Health	International guideline	2	(8)
Biosafety Manual	Boston University	Manual	1	(17)
Laboratory Biosafety Manual	World Health Organization	International guideline	2	(11)
Biosafety in Microbiological and Biomedical Laboratories	Centers for Disease Control and Prevention, National Institutes of Health	Manual	1	(18)
Laboratory activities involving transmissible spongiform encephalopathy causing agents. Risk assessment and biosafety recommendations in Belgium.	Leunda et al.	Journal article	2	(19)
Guidance. Minimise transmission risk of CJD and vCJD in healthcare settings	Department of Health and Social Care, Government of the United Kingdom	National guideline provided by another country	1	(20)
Overview of the BSE risk assessments of the European Commission’s Scientific Steering Committee (SSC) and its TSE/BSE ad hoc Group	Former Scientific Steering Committee of the European Commission	Report	1	(21)
Regulation (EC) No 999/2001 of the European Parliament and of the Council of 22 May (10) laying down rules for the prevention, control and eradication of certain transmissible spongiform encephalopathies	European Commission	International regulation	2	(22)
Directive (2000)/54/EC of the European Parliament and of the Council of 18 September (2000) on the protection of workers from risks related to exposure to biological agents at work (seventh individual directive within the meaning of Article 16(1) of Directive 89/391/EEC)	European Commission	International regulation	1	(23)
Note for guidance on minimising the risk of transmitting animal spongiform encephalopathy agents via human and veterinary medicinal products (EMA/410/01 rev.3) (2011/C 73/01)	European Commission	International regulation	1	(24)

**Figure 3:**
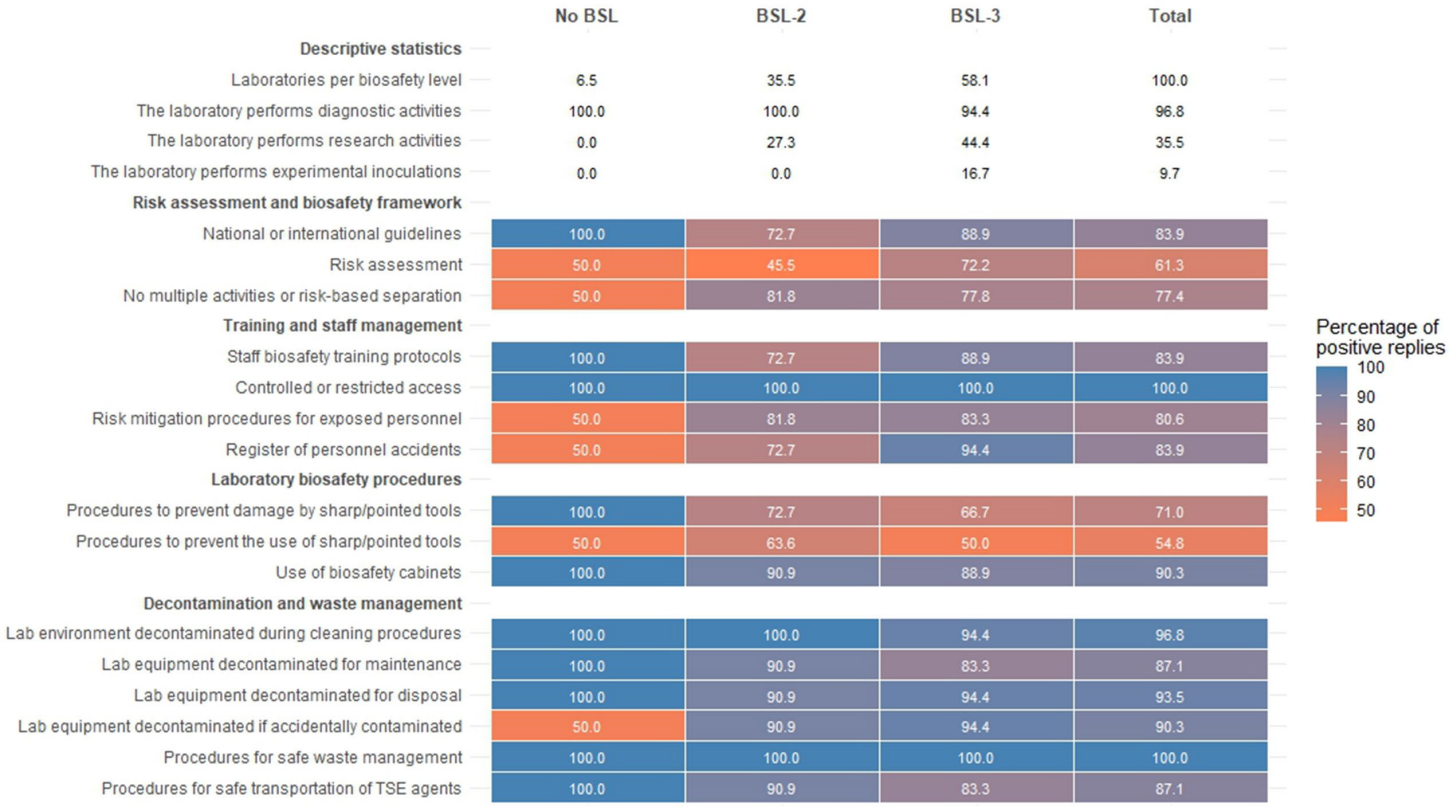
Activities conducted and compliance with biosafety procedures at National Reference Laboratories, stratified by biosafety level (BSL) and by survey item.

A biosafety risk assessment had been carried out in almost two thirds of laboratories (*n* = 19, 61.3%), while the BSL had been determined for almost all (*n* = 29, 93.5%) (Figure 3). BSL-3 was the highest biosafety level applied in 18 NRLs (58.1%), while BSL-2 was the highest in 11 NRLs (35.5%, with 5 of these implementing supplementary measures beyond those mandated for BSL-2). When reporting laboratory BSL, biocontainment facilities, whether dedicated or within mixed-level buildings, were considered equivalent. Only two laboratories (6.5%) had not determined their BSL.

Most NRLs (*n* = 26, 83.9%) were exclusively dedicated to TSE agents. Nearly all laboratories (*n* = 30, 96.8%) performed diagnostic activities, with minimal differences between BSL. Approximately one third (*n* = 11, 35.5%) were involved in research activities, predominantly BSL-3 laboratories, followed by BSL-2 facilities. Only 3 (9.7%) reported conducting experimental inoculations, all of them classified as BSL-3. Three quarters of the laboratories (*n* = 24, 77.4%) conducted the different activities (diagnostics, research, and experimental inoculations) concerning TSEs in separate areas according to their level of risk or did not engage in multiple activities.

Staff biosafety training protocols were widely implemented (*n* = 26, 83.9%), and access to TSE laboratories was restricted to authorised personnel only (*n* = 31, 100.0%). In case of personnel exposure, most laboratories applied specific procedures to mitigate the risk of transmission (*n* = 25, 80.6%) and kept record of the events in a register of personnel accidents (*n* = 26, 83.9%). Over three quarters of NRLs applied specific procedures to prevent the use of sharp or pointed tools or the risks associated with them (*n* = 24, 77.4%). The vast majority of NRLs employed biosafety cabinets (*n* = 28, 90.3%). Compliance with training and staff management procedures was consistently higher or equal in BSL-3 laboratories compared with BSL-2 laboratories.

Specific biosafety procedures for ELISA and western blot methods were available in most facilities conducting diagnostic (*n* = 24; 80.0%) and research (*n* = 9; 81.8%) activities (Figure 2B). However, biosafety procedures specific for pathology and immunohistochemistry were less commonly available in both diagnostics (*n* = 17, 56.7%) and research (*n* = 7, 63.6%) laboratories. Of the three laboratories performing experimental inoculations, two reported employing specific biosafety procedures for this practice (66.7%).

Across all BSL categories, most laboratories implemented measures for the laboratory environment, including surfaces, during cleaning procedures (*n* = 30; 96.8%), as well as for the laboratory equipment in case of maintenance (*n* = 27, 87.1%), disposal (*n* = 29, 93.5%), or accidental contamination (*n* = 28, 90.3%). The totality of NRLs had procedures for safe waste management, and most of them employed procedures for safe transportation of TSE agents (*n* = 27, 87.1%).

Major differences in compliance across BSL categories were observed in domains related to risk assessment and the implementation of biosafety frameworks, staff training and management, and laboratory biosafety procedures (Figure 3). In these areas, compliance was heterogeneous, ranging from 45.5 to 100.0%. However, in most cases, BSL-3 laboratories showed slightly higher compliance than BSL-2 laboratories. In contrast, adherence to decontamination and waste management procedures was high across all BSL categories, with only one item at 50.0% and the remainder between 83.3 and 100.0%.

Full survey results are provided in the Supplementary material.

## 4 Discussion

Despite the overall positive results, the survey revealed that biosafety procedures are applied heterogeneously across the NRLs. Each laboratory demonstrated stronger capacity to apply certain measures and varying degrees of adherence to others. Higher BSL did not consistently correspond to greater compliance with biosafety procedures. However, five BSL-2 laboratories implemented measures exceeding their level requirements, and the survey did not distinguish between biocontainment facilities in dedicated versus mixed-level buildings. These factors may have partially masked the influence of BSL on compliance with biosafety procedures. Previous studies have linked variability in biosafety compliance across laboratory settings to multiple factors, including institutional type, the nature of laboratory activities, staffing and resource availability, geographic and administrative contexts, and the professional experience of personnel (12–15). In this study, all participating NRLs were hosted by government agencies or universities but were distributed across 30 countries. The diversity of institutional arrangements, laboratory functions, and local regulatory contexts in which the NRLs operate, coupled with the fact that biosafety procedures are governed at the national level, likely contributes to differences in how biosafety procedures are implemented.

The high resistance of prions, combined with the dramatic consequences of accidental exposure, requires the implementation of tailored biosafety procedures. Five NRLs did not report the use of biosafety guidelines specifically designed for the safe management of TSE agent; in some cases, they adopted procedures intended for the management of generic pathogens. This highlights the need for wider dissemination of such guidelines. Similarly, some of the laboratories were not exclusively dedicated to TSE agents or did not separate the activities based on their level of risk, thus hindering the proper application of safety procedures. The lack of specific procedures for ELISA, western blot methods, pathology, immunohistochemistry, and experimental inoculations reported by certain laboratories involved in such activities further indicates room for improvement. These activities involve direct manipulation of high-risk tissues, generation of aerosols, and the use of sharp instruments, all of which may increase the likelihood of personnel exposure in the absence of standardised procedures. The implementation of activity-specific procedures could therefore play a key role in reducing exposure risks by ensuring consistent application of containment measures, appropriate use of personal protective equipment, and effective decontamination and waste management.

Other critical findings of the survey related to operational aspects of daily laboratory routine that could be improved with a modest investment of time and resources. Almost a quarter of the laboratories did not implement measures to prevent the use or reduce the risk of damage from sharp or pointed tools. At least one of the reported laboratory accidents was caused by stabbing with a sharp tool (9), which likely represents the major source of risk for laboratory personnel. This highlights the need to avoid the use of such tools wherever possible and to regulate their use when it is unavoidable. Furthermore, it is essential that TSE agents are always handled within safety cabinets, and laboratory equipment should be designed to fit within these cabinets. If this is not feasible, closed-system techniques for sample processing should be employed to minimize the risk of exposure. The implementation of decontamination procedures is also crucial to ensure the safety of the laboratory environment and the equipment.

Each stage of the process of sample management should be clearly defined and regulated by specific operating procedures to ensure the adoption of appropriate practices, minimise the risk of exposure, and respond appropriately to accidents. Training schemes should always be available to ensure that the personnel are familiar with these procedures.

The findings of the survey emphasized the importance of conducting a biosafety risk assessment and determining the level of biocontainment through a systematic review of practices, equipment, and facilities. This allows for the methodical identification and resolution of critical points, the implementation of necessary protective measures for the safety of the staff and the public, and a clear delineation of the activities that can be carried out safely within the laboratory. Despite this, the survey revealed that such assessments were incomplete in the NRLs. Risk assessment is a key biosafety tool. It identifies biological hazards, evaluates the likelihood of exposure and potential consequences, and informs the implementation of control measures. In practice, the role of a formal risk assessment in selecting appropriate mitigation measures may nevertheless be minimised or overlooked. This can result in a biological agent being classified within a generic group of pathogens, with the subsequent implementation of unspecific biosecurity measures that have been envisaged for that group. Such an approach fails to consider the risks associated with the specific characteristics of that agent, including the facilities, handling procedures, and protocols required for its safe management. This can lead to an underestimation of the likelihood (probability) of occurrence and the severity of potential harm. With regard to TSEs, the implementation of an appropriate risk assessment scheme leads to the conclusion that the measures to be implemented by TSE diagnostic laboratories are a combination of those required for biosafety level 2 and 3. Particular focus should be on a few crucial operations (puncture/cutting, surface contamination and aerosol production) to prevent environmental contamination and reduce the risk of exposure and infection for operators. The absence of risk assessment is often not only a technical problem, but also an organisational and cultural one. Risk assessment requires structural support, resource allocation, and the definition of responsibilities, all of which depend on management decisions. A lack of structured, periodically updated risk assessments may indicate limited management involvement in biosafety governance, which could affect the effectiveness of the biological risk management system.

Thanks to the 100% response rate, the survey is unlikely to be affected by sampling biases. Nonetheless, several limitations inherent to the survey method should be acknowledged. Self-reported data are vulnerable to response and social desirability biases, which may lead to overestimation of laboratory compliance with biosafety guidelines. No cross-validation methods were used to verify the accuracy of the responses. The reliance on binary and multiple-choice questions constrained the depth of insight into how and why certain practices are implemented. To mitigate this limitation, an open-ended question was included at the end of the questionnaire to allow participants to provide clarifications or add information they considered relevant. The comments collected offered useful details on the application of BSL procedures, and no major concerns regarding the questionnaire design were raised by respondents. Finally, this study was designed to assess biosafety procedures specifically within NRLs; therefore, the findings cannot be generalised to laboratories outside the NRL network.

The EURL-TSE will further encourage the full implementation of biosafety procedures specific for TSEs agents to address identified gaps and minimise risk to laboratory personnel. While the development of biosafety controls and improvement actions remains the responsibility of national authorities, the EURL-TSE promotes awareness and the exchange of best practices across NRLs. Given the abundance of bibliographical resources and guidelines, future actions of the EURL-TSE will focus on securing access to such resources and enhancing their application across all NRLs. To this end, the EURL-TSE has created a digital repository of guidelines and bibliographical sources on the subject (16). It also initiated biannual virtual meetings to elucidate critical issues emerged from the survey and to encourage the mutual exchange of experiences and good practices between NRL Directors. Each meeting will focus on a specific biosafety topic, with the decontamination of the laboratory environment and equipment being the first topic addressed. Moreover, these meetings will serve as a platform to qualitatively investigate the “root causes” of variability in compliance directly with the NRL Directors. Finally, a follow-up survey will be carried out to assess the effectiveness of the awareness and information-sharing efforts and to monitor progress over time; it will also include questions aimed at investigating precisely the institutional or economic barriers that may affect the laboratory biosafety.

The EURL-TSE will continue to promote the critical review and ongoing enhancement of biosafety procedures across European NRLs. As BSE and other transmissible spongiform encephalopathies persist in Europe, ensuring the safety of those working to detect and respond to these diseases remains crucial.

## Data Availability

The anonymised raw data supporting the conclusions of this article will be made available by the authors, without undue reservation.

## Group member of Colleagues of the European Network of National Reference Laboratories for Transmissible Spongiform Encephalopathies

The list of consortium members is: Alexandra Bastian (Austria), Severine Matthijs (Belgium), Dafina Atanasova (Bulgaria), Karmen Branovic Cakanic (Croatia), Sotiria Georgiadou (Cyprus), Petr Václavek (Czech Republic), Tim Kåre Jensen (Denmark), Katrin Peik (Estonia), Sirkka-Liisa Korpenfelt (Finland), Thierry Baron (France), Christine Fast (Germany), Vaia Palaska (Greece), Peter Malik (Hungary), Stefania Thorgeirsdottir (Iceland), Ann Sharpe (Ireland), Maria Caramelli (Italy), Inga Pigiņka-Vjačeslavova (Latvia), Laura Krivko (Latvia), Marta Lizuma (Latvia), Viktoras Maskaliovas (Lithuania), Susan Chircop (Malta), Lucien van Keulen (Netherlands), Sylvie Benestad (Norway), Miroslaw Polak (Poland), Maria Leonor Orge (Portugal), Igor Djadjovski (Republic of North Macedonia), Florica Barbuceanu (Romania), Daniela Korytarova (Slovakia), Kristina Tekavec (Slovenia), Alba Marín Moreno (Spain), Viktor Ahlberg (Sweden), Torsten Seuberlich (Switzerland).

## References

[1] Prusiner SB. Prions. Proc Natl Acad Sci. (1998). 95:13363–83. doi: 10.1073/pnas.95.23.13363, 9811807 PMC33918

[2] Zerr I Ladogana A Mead S Hermann P Forloni G Appleby BS. Creutzfeldt–Jakob disease and other prion diseases. Nat Rev Dis Primers. (2024). 10:14. doi: 10.1038/s41572-024-00497-y, 38424082

[3] WHO. WHO infection control guidelines for transmissible spongiform encephalopathies: Report of a WHO consultation, Geneva, Switzerland, 23–26 march 1999. No. WHO/CDS/CSR/APH/2000.3. Geneva, Switzerland: (2020). (Accessed October 27, 2025).

[4] Tranulis MA Tryland M. The zoonotic potential of chronic wasting disease—a review. Foods. (2023). 12:824. doi: 10.3390/foods12040824, 36832899 PMC9955994

[5] Cassard H Torres J-M Lacroux C Douet J-Y Benestad SL Lantier F . Evidence for zoonotic potential of ovine scrapie prions. Nat Commun. (2014). 5:5821. doi: 10.1038/ncomms6821, 25510416

[6] Comoy EE Mikol J Luccantoni-Freire S Correia E Lescoutra-Etchegaray N Durand V . Transmission of scrapie prions to primate after an extended silent incubation period. Sci Rep. (2015). 5:11573. doi: 10.1038/srep11573, 26123044 PMC4485159

[7] Alcalde-Cabero E Almazán-Isla J Brandel JP Breithaupt M Catarino J Collins S . Health professions and risk of sporadic Creutzfeldt–Jakob disease, 1965 to 2010. Euro Surveill. (2012). 17:20144. doi: 10.2807/ese.17.15.20144-en 22516047

[8] WOAH . (2023). “Biosafety and biosecurity: Standard for managing biological risk in the veterinary laboratory and animal facilities,” in Manual of Diagnostic Tests and Vaccines for Terrestrial Animals, twelfth edition Paris World Organisation for Animal Health. Available online at: https://www.woah.org/en/what-we-do/standards/codes-and-manuals/terrestrial-manual-online-access/

[9] Brandel J-P Vlaicu MB Culeux A Belondrade M Bougard D Grznarova K . Variant Creutzfeldt–Jakob disease diagnosed 7.5 years after occupational exposure. N Engl J Med. (2020). 383:83–5. doi: 10.1056/NEJMc2000687, 32609989

[10] Denouel A Brandel J-P Peckeu-Abboud L Seilhean D Bouaziz-Amar E Quadrio I . Prospective 25-year surveillance of prion diseases in France, 1992 to 2016: a slow waning of epidemics and an increase in observed sporadic forms. Euro Surveill. (2023). 28:2300101. doi: 10.2807/1560-7917.ES.2023.28.50.2300101 38099349 PMC10831413

[11] WHO. (2020). Laboratory biosafety manual, 4th edition Geneva World Health Organization. Available online at: https://www.who.int/publications/i/item/9789240011311 (Accessed October 27, 2025).

[12] Cong Y Li J Lou D Zhu J Zhang D Cheng D . Analysis of compliance issues and influencing factors in the management of BSL-2 laboratories for pathogenic microorganisms in Lishui, China. Front Bioeng Biotechnol. (2025). 13:1637056. doi: 10.3389/fbioe.2025.1637056, 40861857 PMC12371245

[13] Gillum DR Tran A Fletcher J Vogel KM. Bridging biosafety and biosecurity gaps: DURC and ePPP policy insights from U.S. institutions. Front Bioeng Biotechnol. (2024). 12:1476527. doi: 10.3389/fbioe.2024.1476527, 39398640 PMC11467424

[14] Liu Y Guo Y Li S Liu B Wen J Zhao C. Investigation and analysis of the biosafety awareness of laboratory staff involved in the detection of pathogens in seven provincial Centers for Disease Control and Prevention in China. Biosaf Heal. (2021). 3:224–9. doi: 10.1016/j.bsheal.2021.07.001

[15] Niu P Sun Z Zhang R Zhao Y Tian F Cheng P . The state of biosafety across China’s CDC microbiology laboratories: insights from a nationwide survey (2021–2023). Front Public Health. (2024). 12:1436503. doi: 10.3389/fpubh.2024.1436503, 39157525 PMC11327048

[16] EURL-TSE. (2023). Official website of the European Reference Laboratory for Transmissible Spongiform Encephalopathies. Available online at: https://www.eurl-tse.eu/ (Accessed October 27, 2025).

[17] Boston University. (2022). “Appendix H: prion research/Creutzfeldt-Jacob disease (CJD) guidelines,” in Biosafety manual, (Boston, MA: Boston University Office of Research). Available online at: https://www.bu.edu/research/ethics-compliance/safety/biological-safety/ibc/resources/biosafety-manual/ (Accessed October 27, 2025).

[18] CDC. (2020). Biosafety in microbiological and biomedical laboratories 6th. Atlanta: Centers for Disease Control and Prevention, National Institutes of Health. Available online at: https://www.cdc.gov/labs/BMBL.html (Accessed October 27, 2025).

[19] Leunda A Van Vaerenbergh B Baldo A Roels S Herman P. Laboratory activities involving transmissible spongiform encephalopathy causing agents. Prion. (2013) 7:420–33. doi: 10.4161/pri.26533, 24055928 PMC3904386

[20] UK. (2012). Guidance. Minimise transmission risk of CJD and vCJD in healthcare settings. Department of Health and Social Care, Government of the United Kingdom. Available online at: https://www.gov.uk/government/publications/guidance-from-the-acdp-tse-risk-management-subgroup-formerly-tse-working-group (Accessed October 27, 2025).

[21] EC. (2003). WHO infection control guidelines for transmissible spongiform encephalopathies: Report of a WHO consultation, 23–26 march 1999. No. WHO/CDS/CSR/APH/2000.3. Geneva, Switzerland: World Health Organization Available online at: https://food.ec.europa.eu/system/files/2020-12/sci-com_ssc_out364_en.pdf (Accessed October 27, 2025).

[22] WHO. (2001). Regulation (EC) No 999/2001 of the European Parliament and of the Council of 22 May 2001 laying down rules for the prevention, control and eradication of certain transmissible spongiform encephalopathies. Available online at: http://data.europa.eu/eli/reg/2001/999/2023-01-01

[23] EC. (2000). Directive 2000/54/EC of the European Parliament and of the Council of 18 September 2000 on the protection of workers from risks related to exposure to biological agents at work (seventh individual directive within the meaning of Article 16(1) of Directive. Available online at: http://data.europa.eu/eli/dir/2000/54/2020-06-24 (Accessed October 27, 2025).

[24] EC. (2011). Note for guidance on minimising the risk of transmitting animal spongiform encephalopathy agents via human and veterinary medicinal products (EMA/410/01 rev.3). Available online at: https://eur-lex.europa.eu/legal-content/EN/TXT/?uri=CELEX:52011XC0305(04) (Accessed October 27, 2025).

