# Supplementary material for: State of the art in biosafety at the European National Reference Laboratories for Transmissible Spongiform Encephalopathies
Source: Front Public Health. 2026 Feb 10;13:1733350. doi: 10.3389/fpubh.2025.1733350 (PMC12929439; doi:10.3389/fpubh.2025.1733350)
Supplement: Supplementary file 1 [file Table_1.docx]

Supplementary Material

**Aggregated survey results**

**Table 1.** Survey questions proposed to the directors of National Reference Laboratories for Transmissible Spongiform Encephalopathies and full survey results.

| **Question** | **Answers** | **n** | **%** |
| --- | --- | --- | --- |
| Are there any specific national guidelines for safely working with TSE agents? | Yes | 19 | 61.3 |
|  | No | 12 | 38.7 |
| If not, do you refer to specific international guidelines? | Yes | 7 | 58.3 |
|  | No | 5 | 41.7 |
| Have you carried out a risk assessment in the laboratory/laboratories that deal with TSE agents? | Yes | 19 | 61.3 |
|  | No | 12 | 38.7 |
| Have you determined the level of biocontainment of the laboratory/laboratories that deal with TSE agents (BSL-2, BSL-3, or any other classification system)? | Yes | 29 | 93.5 |
|  | No | 2 | 6.5 |
| If yes, which is the level assigned to the laboratory/laboratories? [open-ended question] | BSL-2 | 11 | 37.9 |
|  | BSL-3 | 18 | 62.1 |
| Which activities are performed in the laboratory/laboratories that deal with TSE agents? | Diagnosis | 30 | 96.8 |
|  | Research | 11 | 35.5 |
|  | Experimental inoculations | 3 | 9.7 |
| If multiple activities (diagnosis, research, etc.) are carried out in the laboratory/laboratories, are the different activities conducted in separate areas according to their level of risk? | Yes | 6 | 19.3 |
|  | No | 7 | 22.6 |
|  | No multiple activities take place in the laboratory | 18 | 58.1 |
| Are the laboratory/laboratories solely dedicated to TSE agents activities, or are there additional unrelated activities being carried out within the same laboratory? | The laboratory is solely dedicated to TSE agents activities | 26 | 83.9 |
|  | Activities not related to TSE agents are carried out in the laboratory | 5 | 16.1 |
| Have you implemented biosafety training protocols for staff dealing with TSEs? | Yes | 26 | 83.9 |
|  | No | 5 | 16.1 |
| Do you impose controlled or restricted access (only authorised personnel) to the TSE laboratory/laboratories? | Yes | 31 | 100.0 |
|  | No | 0 | 0.0 |
| Do you have specific biosafety procedures available for ELISA/Western Blot? | Yes | 24 | 77.4 |
|  | No | 7 | 22.6 |
| Do you have specific biosafety procedures available for pathology/immunohistochemistry? | Yes | 17 | 54.8 |
|  | No | 14 | 45.2 |
| Do you have specific biosafety procedures available for experimental inoculations? | Yes | 5 | 16.1 |
|  | No | 26 | 83.9 |
| Do you have any specific procedures in place to prevent the use of sharp or pointed tools? | Yes | 17 | 54.8 |
|  | No | 14 | 45.2 |
| Do you have established procedures in place to prevent damage caused by the use of sharp or pointed tools? | Yes | 22 | 71.0 |
|  | No | 9 | 29.0 |
| Do you employ biosafety cabinets? | Yes | 28 | 90.3 |
|  | No | 3 | 9.7 |
| Are measures implemented to decontaminate the laboratory environment, including surfaces, during cleaning procedures? | Yes | 30 | 96.8 |
|  | No | 1 | 3.2 |
| Are there procedures in place to decontaminate the laboratory equipment in case it needs maintenance? | Yes | 27 | 87.1 |
|  | No | 4 | 12.9 |
| Are there procedures in place to decontaminate the laboratory equipment in the event of its disposal? | Yes | 29 | 93.5 |
|  | No | 2 | 6.5 |
| Are there procedures in place to decontaminate the laboratory equipment in case of accidental contamination? | Yes | 28 | 90.3 |
|  | No | 3 | 9.7 |
| Are there specific procedures to mitigate the risk in the event of personnel exposure? | Yes | 25 | 80.6 |
|  | No | 6 | 19.4 |
| Are there specific procedures for safe waste management? | Yes | 31 | 100.0 |
|  | No | 0 | 0.0 |
| Are there specific procedures for the safe transportation of TSE agents? | Yes | 27 | 87.1 |
|  | No | 4 | 12.9 |
| Is there a register of personnel accidents? | Yes | 26 | 83.9 |
|  | No | 5 | 16.1 |
| Please provide any other comments you deem useful [open-ended question] | - | - | - |

**Acknowledgements**

The Authors wish to thank the Network contact persons for their willingness to participate in the survey. Contact persons are listed in alphabetical order by country: Alexandra Bastian (Austria), Severine Matthijs (Belgium), Dafina Atanasova (Bulgaria), Karmen Branovic Cakanic (Croatia), Sotiria Georgiadou (Cyprus), Petr Václavek (Czech Republic), Tim Kåre Jensen (Denmark), Katrin Peik (Estonia), Sirkka-Liisa Korpenfelt (Finland), Thierry Baron (France), Christine Fast (Germany), Vaia Palaska (Greece), Peter Malik (Hungary), Stefania Thorgeirsdottir (Iceland), Ann Sharpe (Ireland), Maria Caramelli (Italy), Inga Pigiņka-Vjačeslavova (Latvia), Laura Krivko (Latvia), Marta Lizuma (Latvia), Viktoras Maskaliovas (Lithuania), Susan Chircop (Malta), Lucien van Keulen (Netherlands), Sylvie Benestad (Norway), Miroslaw Polak (Poland), Maria Leonor Orge (Portugal), Igor Djadjovski (Republic of North Macedonia), Florica Barbuceanu (Romania), Daniela Korytarova (Slovakia), Kristina Tekavec (Slovenia), Alba Marín Moreno (Spain), Viktor Ahlberg (Sweden), Torsten Seuberlich (Switzerland).
